# Grand Rounds: Multidisciplinary Management of the Patient With Metastatic Colorectal Adenocarcinoma

**DOI:** 10.6004/jadpro.2015.6.2.6

**Published:** 2015-03-01

**Authors:** Steve A. Malangone, Hitendra Patel, Sandra E. Kurtin, Hina Arif Tiwari, Emad Elquza

**Affiliations:** University of Arizona Cancer Center, Tucson, Arizona

**Grand Rounds: Multidisciplinary Management of the Patient With Metastatic Colorectal Adenocarcinoma**

A continuing education article for nurse practitioners, physician assistants, clinical nurse specialists, advanced degree nurses, oncology and hematology nurses, pharmacists, and physicians.

**Release date:** March 15, 2015

**Expiration date:** March 15, 2016

**Expected time to complete this activity as designed:** .50 hours

**Meniscus Educational Institute**

100 Overlook Center

2nd Floor

Princeton, NJ 08540

Voice: 609-246-5000

Fax: 609-449-7969

E-mail: mthompson@meniscusedu.com

**Journal of the Advanced Practitioner in Oncology**

37 Main Street

Cold Spring Harbor, NY 11724

Voice: 631-692-0800

Fax: 631-692-0805

E-mail: claudine@harborsidepress.com

© *2015, Meniscus Educational Institute. All rights reserved.*

## Faculty

**Steve A. Malangone, NP-C,** University of Arizona Cancer Center

**Hitendra Patel, MD,** University of Arizona Cancer Center

**Sandra E. Kurtin, RN, MS, AOCN®, ANP-C,** University of Arizona Cancer Center

**Hina Arif Tiwari, MD,** University of Arizona Cancer Center

**Emad Elquza, MD,** University of Arizona Cancer Center

## Activity Rationale and Purpose

It has been clearly demonstrated that a high-quality and efficient cancer care system requires effective multidisciplinary teams that collaborate to provide patient-centered care. The current case report provides an example of the effective management of metastatic colorectal cancer through the use of a multidisciplinary tumor conference (MTC) to provide coordinated care throughout the disease continuum. The case provides a template for how other multidisciplinary teams can overcome some of the barriers to effective implementation of this type of care. The advanced practitioner plays a key role in the multidisciplinary management of these patients, and whenever possible, should be engaged in the MTC.

## Intended Audience

The activity’s target audience will consist of nurse practitioners, physician assistants, clinical nurse specialists, advanced degree nurses, oncology and hematology nurses, pharmacists, and physicians.

## Learning Objectives

After completing this educational activity, participants should be able to:

1. List the benefits of a multidisciplinary team-based approach to the management of metastatic colorectal cancer (CRC)
2. Describe the members of the multidisciplinary team, including advanced practitioners, required to provide optimal care for CRC patients
3. Review current approaches at their own institutions to assess how well multidisciplinary team-based care is being implemented there
4. Identify barriers and opportunities at their own institutions for the implementation of a team-based approach to CRC management
5. Develop a plan for the development and coordination of multidisciplinary teams in CRC care

## Continuing Education

**Statement of Credit—Participants who successfully complete this activity (including the submission of the post-test and evaluation form) will receive a statement of credit.**

**Physicians.** The Meniscus Educational Institute is accredited by the Accreditation Council for Continuing Medical Education (ACCME) to provide continuing medical education for physicians.

The Meniscus Educational Institute designates this journal article (2015-002-00-MJ) for a maximum of 0.50 AMA PRA Category 1 Credits™. Physicians should claim only the credit commensurate with the extent of their participation in the activity.

**Nurses.** This activity (2015-002-00-NJ) for 0.50 contact hours is provided by the Meniscus Educational Institute.

The Meniscus Educational Institute is accredited as a provider of continuing nursing education by the American Nurses Credentialing Center’s Commission on Accreditation.

Provider approved by the California Board of Registered Nursing, Provider No. 13164, for 0.50 contact hours.

**Pharmacists.** The knowledge-based accredited education lectures are intended for pharmacists involved in the care of cancer patients. This educational activity is sponsored by the Meniscus Educational Institute.

The Meniscus Educational Institute is accredited by the Accreditation Council for Pharmacy Education (ACPE) as a provider of continuing pharmacy education. The ACPE Universal Activity Number assigned to this program, for 0.50 contact hours, is 0429-0000-15-002-H01-P.

## Financial Disclosures

All individuals in positions to control the content of this program (eg, planners, faculty, content reviewers) are expected to disclose all financial relationships with commercial interests that may have a direct bearing on the subject matter of this continuing education activity. Meniscus Educational Institute has identified and resolved all conflicts of interest in accordance with the MEI policies and procedures. Participants have the responsibility to assess the impact (if any) of the disclosed information on the educational value of the activity.

**Faculty**

**Steve A. Malangone, NP-C,** has received honoraria from Taiho Oncology.

**Hitendra Patel, MD,** has nothing to disclose.

**Sandra E. Kurtin, RN, MS, AOCN®, ANP-C,** has nothing to disclose.

**Hina Arif Tiwari, MD,** has nothing to disclose.

**Emad Elquza, MD,** has nothing to disclose.

**Lead Nurse Planner**

**Rita Wickham, PhD, RN, AOCN®,** has received honoraria from Genentech.

**Planners**

**Jeannine Coronna** has nothing to disclose.

**Claudine Kiffer** has nothing to disclose.

**Terry Logan, CHCP,** has nothing to disclose.

**Molly Thompson** has nothing to disclose.

**Pamela Hallquist Viale, RN, MS, CNS, ANP,** has nothing to disclose.

**Content Reviewers**

**Glenn Bingle, MD, PhD, FACP,** has nothing to disclose.

**Christopher J. Campen, PharmD, BCPS, BCOP,** has owned stock from AbbVie.

**Wendy J. Smith, ACNP, AOCN®,** has nothing to disclose.

## Disclaimer

This activity has been designed to provide continuing education that is focused on specific objectives. In selecting educational activities, clinicians should pay special attention to the relevance of those objectives and the application to their particular needs. The intent of all Meniscus Educational Institute educational opportunities is to provide learning that will improve patient care. Clinicians are encouraged to reflect on this activity and its applicability to their own patient population.

The opinions expressed in this activity are those of the faculty and reviewers and do not represent an endorsement by Meniscus Educational Institute of any specific therapeutics or approaches to diagnosis or patient management.

## Product Disclosure

This educational activity may contain discussion of published as well as investigational uses of agents that are not approved by the US Food and Drug Administration. For additional information about approved uses, including approved indications, contraindications, and warnings, please refer to the prescribing information for each product.

## How to Earn Credit

To access the learning assessment and evaluation form online, visit www.meniscusce.com

**Statement of Credit:** Participants who successfully complete this activity (including scoring of a minimum of 70% on the learning assessment and complete and submit the evaluation form with an E-mail address) will be able to download a statement of credit.

## CASE STUDY

A 60-year-old man initially presented with rectal bleeding in March 2006. He underwent a colonoscopy, which revealed a rectosigmoid mass, and the biopsy specimen confirmed adenocarcinoma.

In May 2006, the patient underwent planned low anterior resection. During the procedure, he was found to have a metastatic lesion in the left hepatic lobe. Hepatobiliary surgery was consulted, and a left lateral liver resection was performed at the time of the initial operation. Complete surgical pathology revealed T3, N2, M1, with 11 of 28 lymph nodes positive for disease.

The patient then went on to receive postoperative treatment with fluorouracil (5-FU), leucovorin, and oxaliplatin (FOLFOX) and bevacizumab (Avastin). His treatment was interrupted due to sudden cardiac arrest requiring resuscitation measures for ventricular fibrillation. He had an implanted defibrillator placed and resumed treatment with 5-FU/oxaliplatin and bevacizumab chemotherapy. He was then changed to 5-FU and weekly oxaliplatin with concurrent radiotherapy. This treatment was completed in October 2006. Afterward, the patient completed an additional 4 cycles of FOLFOX/bevacizumab, with the last dose given in December 2006.

The patient remained on surveillance without evidence of tumor recurrence for more than 3.5 years, until a September 2010 CT scan revealed a segment 7 hepatic lesion. He was treated with 3 cycles of 5-FU/irinotecan (FOLFIRI).

His case was discussed in the multidisciplinary colorectal tumor conference. He was not deemed a candidate for further hepatic resection due to the location of the lesion and prior extensive resection. Various approaches to therapy were discussed, and he was taken for exploratory laparotomy with intraoperative ultrasonography of the liver and radiofrequency ablation of a single lesion in segment 7. He was treated with an additional 2 months of FOLFIRI through April 2011.

**Figure 1:**
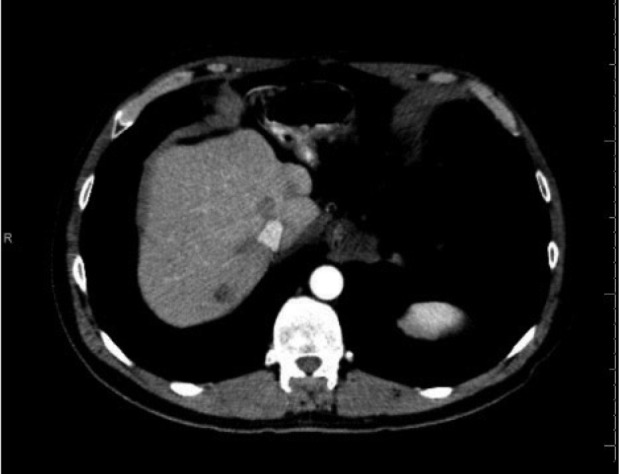
September 2011 CT scan of the chest, abdomen, and pelvis reveals an enlargement of a previously demonstrated segment 7 hepatic lesion.

**Figure 2:**
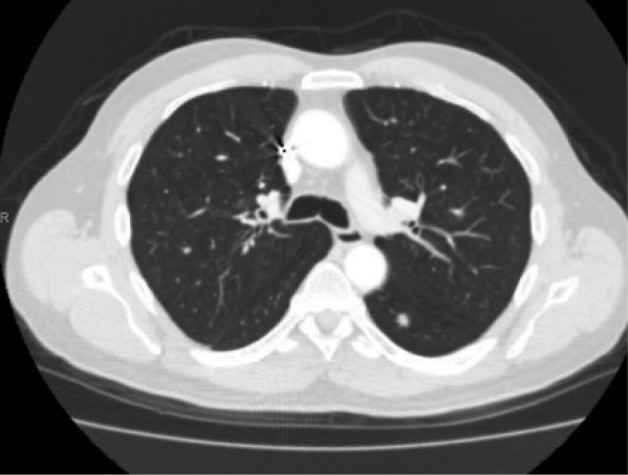
September 2011 CT scan of the chest, abdomen, and pelvis reveals a 7-mm left lower-lobe pulmonary nodule.

**Figure 3:**
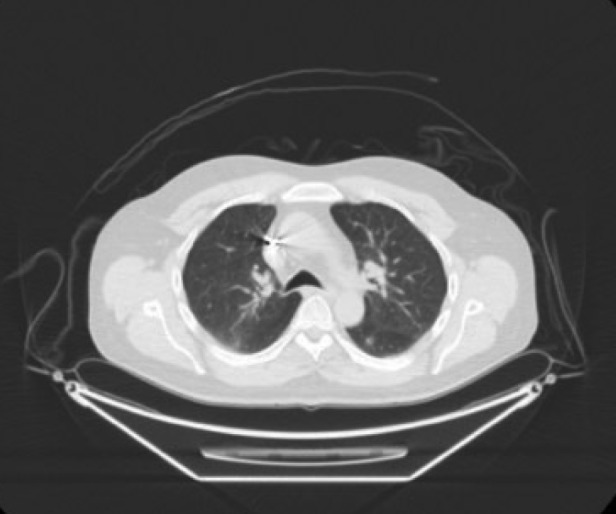
August 2012 CT scan shows excellent treatment response after 7 cycles of capecitabine/oxaliplatin/cetuximab.

In September 2011, a CT scan of the chest, abdomen, and pelvis revealed enlargement of a previously demonstrated segment 7 hepatic lesion and a 7-mm left lower-lobe pulmonary nodule (Figures 1 and 2). CT-guided biopsy of the lung lesion confirmed metastatic colorectal cancer. At that time, *KRAS* testing was requested, which revealed no *KRAS* mutation. The patient was treated with capecitabine/oxaliplatin/cetuximab (Erbitux) for 7 cycles. In August 2012, a follow-up PET/CT scan revealed a complete metabolic response (Figure 3). He was then transitioned to single-agent weekly cetuximab.

**Figure 4:**
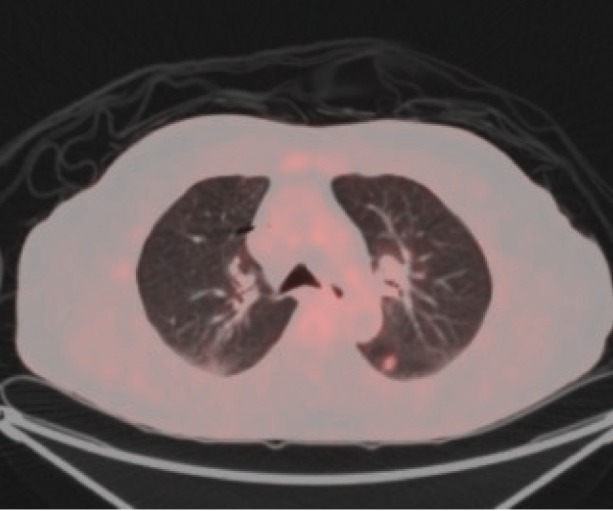
April 2013 PET-CT scan reveals increased metabolic activity in the size of the left lower-lobe lung lesion.

In April 2013, a PET-CT scan revealed an increase in metabolic activity and size of the left lower-lobe lung lesion (Figure 4). His case was discussed at the colorectal Multidisciplinary Tumor Conference; based on this discussion, he was referred to radiation oncology for stereotactic body radiation therapy (SBRT) of the pulmonary nodule, which was completed at the end of May 2013. A PET/CT scan obtained in September 2013 revealed evidence of a good response to the left pulmonary lesion and no evidence of new or recurrent disease.

In December 2013, a subsequent PET/CT scan revealed a single lesion in the liver at the dome and no disease elsewhere. This lesion was present in 2012 and disappeared with treatment. Again, this case was discussed at the colorectal multidisciplinary tumor conference, and the patient was referred for radiofrequency ablation to this lesion, which was completed in February 2014.

A follow-up PET-CT scan in mid-March 2014 revealed a good response to therapy, with no evidence of viable tumor. Unfortunately, PET/CT completed in late June 2014 revealed two new fluorodeoxyglucose (FDG)-avid lesions in the liver in segments 6 and 7. Carcinoembryonic antigen (CEA) also was elevated (6.3 ng/mL, compared with 4.7 ng/mL in March 2014).

The case was once again discussed at the colorectal Multidisciplinary Tumor Conference. The segment 7 lesion was identified as recurrence at the periphery of a previously treated lesion. The patient was referred back to interventional radiology for consideration of additional liver-directed therapy. In addition, given the rising CEA level and the clinical picture suggestive of systemic tumor progression, the patient was restarted on systemic chemotherapy with irinotecan/panitumumab (Vectibix).

An estimated 50,310 of the 136,830 Americans diagnosed with colorectal cancer will die annually of the disease (Siegel, Ma, Zou, & Jemal, 2014). The majority of these deaths are associated with metastatic disease (Siegel et al., 2014). Outcomes for patients with unresectable metastatic disease treated with chemotherapy alone continue to improve. Recent data from the Cancer and Leukemia Group B/Southwest Oncology Group 80405 trial demonstrated average overall survival rates for patients with unresectable metastatic colorectal cancer of more than 29 months and 5-year survival of 10% when treated with current chemotherapy options (Venook et al., 2014).

## MULTIDISCIPLINARY APPROACHES TO TREATMENT

**Surgical Resection**

The group of patients presenting with metastatic colorectal adenocarcinoma can be subdivided into two groups: those with oligometastatic disease and those with extensive/diffuse metastases. In the group of patients with oligometastatic disease to the liver, locoregional treatment with resection of all gross disease has been described extensively in the literature and has become the standard of care, with 5-year survival rates in some series exceeding 50% (Khatri, Petrelli, & Belghiti, 2005).

Although outcomes of this aggressive surgical approach are encouraging, only 15% to 20% of patients with colorectal cancer and liver metastases are surgical resection candidates (Cirocchi et al., 2012). Furthermore, even in carefully selected patients, recurrence rates for patients after hepatic resection of colorectal metastases are high, approaching 75% (Nordlinger et al., 2008). At the time of tumor recurrence, there remains the potential for additional resection if technically feasible, and the disease burden remains limited. Unfortunately, many of these patients may not be candidates at the time of tumor recurrence due to limited residual healthy liver tissue, comorbidities, or the location of the lesion.

**Locoregional Therapies**

Prior hepatic resection often limits additional resection, and the practical management of patients post hepatic resection often involves a coordinated effort combining both locoregional and systemic therapies. In the setting of oligometastatic disease in both the liver and lungs, many clinicians believe additional locoregional therapies have the potential to improve outcomes in selected patients. Shah and colleagues (2006) reported that disease-free and overall survival rates after initial locoregional therapy were 19.8 and 87 months, respectively.

When surgical resection is not medically appropriate, additional locoregional therapies may be considered in carefully selected patients with limited metastatic disease. They include hepatic arterial infusion, transarterial embolization of chemotherapy-eluting beads or radioactive (yttrium-90 [^90^Y]) microbeads, radiofrequency ablation, microwave ablation, and conformal (stereotactic) external-beam radiation (National Comprehensive Cancer Network, 2014).

Although many retrospective series have been published, limited randomized data are available to support the universal application of cytoreductive/locoregional therapies in the management of metastatic colorectal cancer (Cirocchi et al., 2012). Primarily, selection bias and the heterogeneity of both intervention modality and disease presentation limit the generalizability of data from published studies (Cirocchi et al., 2012). The data from these series are not of sufficient quality to draw conclusions, but the potential benefit may be evidenced in carefully selected patients.

**Chemotherapy**

Additionally, systemic chemotherapy plays a significant role in the management of metastatic colon cancer. Chemotherapy is the only modality capable of addressing the problem of systemic disease. Chemotherapy also is important in the perioperative setting, as both an adjuvant treatment and for the purpose of neoadjuvant downstaging.

In the European Organisation for Research and Treatment of Cancer Intergroup trial 40983, Nordlinger et al. (2008) demonstrated a 7.3% 3-year progression-free survival advantage with the addition of perioperative chemotherapy (35% vs. 28.1%). These results support the concept that chemotherapy plays a key part in the adjuvant and neoadjuvant settings of locoregional therapies and may play a role in the eradication of micrometastatic disease.

Chemotherapeutic options have expanded significantly, with advancements in both targeted and cytotoxic agents. Cytotoxic agents include 5-FU (or the oral fluoropyrimidine capecitabine), oxaliplatin, and irinotecan combinations. Targeted agents include vascular endothelial growth factor inhibitors (bevacizumab and ziv-aflibercept [Zaltrap]), epidermal growth factor receptor inhibitors (cetuximab, panitumumab for *KRAS*, *BRAF*, and *NRAS* wild-type tumors), and multitargeted tyrosine kinase inhibitors (such as regorafenib [Stivarga]).

## THE MULTIDISCIPLINARY TUMOR CONFERENCE

The nature of multimodality approaches requires effective communication among multiple disciplines. One way to enhance such communication is the multidisciplinary tumor conference (MTC). At the conference, members of various disciplines involved in site-specific cancer care meet in a designated location and via video/teleconferencing. The patient case is presented with joint review of actual medical data, including history, radiographic and endoscopic images, and pathology slides. Each member of the team provides input in the assessment of the case, and available treatment modalities are discussed, focusing on the development of an individualized consensus approach for the patient in accordance with current best practice (Pawlik et al., 2008).

The implementation of an MTC has been associated with improved patient outcomes. For example, MTCs have been demonstrated to reduce the time between diagnosis and treatment (Wright et al., 2007). Additional positive outcomes in planning, survival, patient satisfaction, and in clinician satisfaction in cooperation/communication have been established as well (Wright, DeVito, Langer, & Hunter, 2007). Most important, associations with improved overall survival have been observed in centers that include multidisciplinary management in the setting of a formal MTC (Sainsbury, Haward, Rider, Johnston, & Round, 1995).

## DESCRIPTION OF THE SINGLE-CENTER EXPERIENCE

**Format**

At our institution, the colorectal MTC meeting occurs weekly at 7 am in a conference room. The room has a round table, which allows for face-to-face discussion. Members from radiology, interventional radiology, medical oncology, surgical oncology, radiation oncology, gastroenterology, and pathology attend regularly. Cases are added throughout the week, and a roster is distributed the day before. Each member of the conference can present cases for review.

**Information Reviewed**

Typically, a case will be presented for the purpose of reviewing diagnostic imaging and pathology. After a brief presentation of the patient case, pertinent images are reviewed on a large projected screen at the front and center of the conference room. These photos are often compared with those from prior studies. Review of imaging may clarify the location of the lesion, response to therapy, and potential sites for biopsy and can be used to assess the potential for surgical, interventional, and radiation approaches to therapy.

Clinical pathology is also present, and actual slides are reviewed and projected onto the big screen as well. A joint review of imaging and pathology may clarify diagnosis and staging as well as define the goals of therapy. Often, discussions ensue, creating opportunities for educational dialog across disciplines.

In clinical case review, helpful information on each case is shared. For example, surgeons can discuss findings during the operation, gastroenterology can describe findings from endoscopy studies and share video and endoscopic retrograde cholangiopancreatography images from procedures. Additional pertinent information can be presented, such as patient tolerance to therapy or which systemic options remain available after locoregional interventions.

## THE ROLE OF THE ADVANCED PRACTITIONER IN THE MTC

Advanced practitioners (APs) at our center work in a blended format, with a mix of clinics— some shared and some independent. Attendance at the MTC provides a great way for the AP to expand his or her knowledge regarding multidisciplinary management. Furthermore, it allows the AP an opportunity to present cases and actively participate in collaborative discussion. The AP can provide clinical context unique to his or her perspective, which may improve and individualize care. In many cases, the AP is in more frequent personal contact with the patient, providing a perspective that clearly influences both goals and therapy. Finally, information received during the conference may provide a basis for educating the patient regarding therapeutic options and medical decision-making.

**Outcomes**

At our center, the outcome of discussion at the MTC often impacts the plan of patient care. For example, for a patient with oligometastatic disease of the liver, the sequencing of therapy may be discussed in relation to patient characteristics. In the event of clearly resectable liver lesions, the consensus plan may be for up-front resection followed by adjuvant chemotherapy. On the contrary, in the event of disease that is unlikely to be amenable to R0 resection up-front, the panel may discuss downstaging, with liver-directed treatment and chemotherapy in the adjuvant setting followed by reimaging to downstage metastatic sites. The potential risks and benefits of various approaches can be discussed with respect to specific patient characteristics. As a result of direct input from experts in each modality, an ultimate evidence-based individualized plan of care can be determined.

Individual review of cases results in unified management of complex cases. Each member can present cases whenever clarification is needed with respect to diagnosis, response to therapy, goals of therapy, or alternative modalities of therapy. A joint review clarifies data and goals and reduces delays in delivery of complex, multidisciplinary care.

Review by MTC promotes individualized management of each patient, with the patient at the center. Cirocchi et al. (2012) reported selection bias and heterogeneity of patient characteristics as major limiting factors in preventing the generalizability of data with respect to locoregional management in the metastatic setting. Although these limitations are accurate, we believe an individually tailored plan can result in significant clinical benefit.

In our case study, the case details were presented and reviewed at each major transition at the MTC. Careful selection of appropriate management and clearly communicated and jointly reviewed diagnostic data in the setting of the MTC doubtlessly contributed to improved outcomes for this patient, who remains alive 8 years from the diagnosis of metastatic disease. This outcome is in stark contrast to current data, indicating an average overall survival of 29 months and a 5-year survival of 10% for patients treated with current chemotherapy options alone (Venook et al., 2014).

The patient in this case with stage IV colon cancer currently has good activity, performance status, and quality of life. He has also experienced a life-threatening event (sudden cardiac arrest while on 5-FU). Although rare, cardiotoxicity to 5-FU is possible and cannot be excluded as a possible cause in this case. Despite this limitation, which threatened to limit his therapeutic options, he went on to receive effective management through an MTC-mediated coordinated blend of systemic, surgical, and interventional approaches.

Ultimately, he has spent less than 50% of the past 8 years on active therapy in the setting of a disease with classic overall survival rates much lower than his. This case provides an example of how multidisciplinary oncology care can promote optimal outcomes in carefully selected patients. The AP plays a key role in the multidisciplinary management of these patients and, whenever possible, should be engaged in the MTC.
